# A case of chronic kidney disease with refractory periodic vomiting and hypertension in a pediatric patient

**DOI:** 10.1007/s13730-024-00905-y

**Published:** 2024-07-11

**Authors:** Yasuyo Kashiwagi, Hironobu Okuno, Satoko Nishida, Hiroki Ishii, Gaku Yamanaka

**Affiliations:** 1 Department of Pediatrics and Adolescent Medicine, Tokyo Medical University, 6-7-1 Nishishinjuku, Shinjuku-Ku, Tokyo, 160-0023 Japan; 2 Department of Pediatrics, Ogikubo Hospital, Tokyo, Japan

**Keywords:** Chronic kidney disease (CKD), Refractory periodic vomiting, Hypertension, Cytokine, Chemokine

## Abstract

Patients with chronic kidney disease (CKD) sometimes experience gastrointestinal symptoms, such as nausea and vomiting. In addition, hypertension and CKD are closely linked, and sustained hypertension in children is associated with the progression of CKD, leading to early cardiomyopathy and premature atherosclerosis. These symptoms substantially affect the quality of daily life of CKD patients, and particularly in children with CKD, they may cause growth retardation. Therefore, establishing effective management methods to alleviate these symptoms is very important. Here, we report a case of a male patient who was born at 34 weeks of gestation weighing 1400 g. At birth, abdominal ultrasonography displayed left multicystic dysplastic kidney. From 6 months after birth, he was frequently hospitalized owing to refractory periodic vomiting. At 9 months old, he was diagnosed as having stage 3a CKD, and at 20 months old, he presented with stage 2 high blood pressure. In Japan, the uremic toxin adsorbent AST-120 and angiotensin-converting enzyme inhibitor-I (ACE-I) are not strongly recommended for CKD patients. However, after the patient underwent combination therapy of AST-120 and ACE-I, his frequent hospitalizations for refractory periodic vomiting ceased, and his blood pressure decreased. These results indicate that AST-120 and ACE-I are effective for refractory periodic vomiting and hypertension associated with CKD. The patient’s height, weight, and mental development are catching up smoothly. The cause of the patient’s refractory periodic vomiting remains unclear. However, his impaired kidney function owing to congenital anomalies of the kidney and urinary tract may have caused the refractory periodic vomiting and dehydration. The production of uremic toxins in CKD patients is thought to lead to the secretion of proinflammatory cytokines into the circulation. However, our patient had low serum levels of proinflammatory cytokines, and his serum levels of the chemokines CX3CL1 and CCL2 decreased with age, together with improvement in his clinical course. Therefore, some specific chemokines might be diagnostic and/or prognostic biomarkers of CKD.

## Introduction

Patients with chronic kidney disease (CKD) may experience a variety of symptoms, including gastrointestinal (constipation, nausea, vomiting, and diarrhea), psychological (anxiety and sadness), neurological (lightheadedness, headache, and numbness), cardiopulmonary (shortness of breath and edema), and dermatological (pruritus and dry skin) symptoms, pain (muscle cramps, chest pain, and abdominal pain), sexual dysfunction, sleep disorders, and fatigue [1]. These symptoms influence the patients’ quality of daily life and may cause growth retardation, particularly in children with CKD. Therefore, establishing effective management methods to alleviate these symptoms is very important.

The uremic toxin adsorbent AST-120 was approved in Japan in 1991 for the purpose of reducing uremic toxin levels. It is useful for prolonging the time to the initiation of hemodialysis therapy and for improving uremic symptoms in patients with CKD. AST-120 has subsequently been approved in other countries (Republic of Korea in 2005 and Republic of the Philippines in 2010) [2].

Hypertension and CKD are closely linked, and hypertension is detected in more than 50% of pediatric patients with CKD [3]. Sustained hypertension in children leads to a worsening of renal function and end-organ damage, including early cardiomyopathy and premature atherosclerosis [3]. Hypertension in CKD is caused by multiple factors, such as reduced nephron mass, increased sodium retention, extracellular volume expansion, sympathetic nervous system overactivity, hormone activation, including the renin–angiotensin–aldosterone system, and endothelial dysfunction [4]. Angiotensin-converting enzyme inhibitor-I (ACE-I) is one of the main blood pressure regulators used clinically around the world. However, it has been reported that ACE-I may cause acute kidney injury in CKD patients [5]. In Japan, AST-120 and ACE-I are not strongly recommended for children with CKD, as stated in the Japanese Evidence-Based Clinical Practice guidelines for CKD 2023 [6].

Here, we report a case of a patient with stage 3a CKD associated with unilateral left multicystic dysplastic kidney (MCDK). The patient had congenital anomalies of the kidney and urinary tract (CAKUT) owing to left MCDK. He underwent frequent hospitalizations owing to refractory periodic vomiting from 6 months of age. At 20 months old, the patient developed stage 2 high blood pressure.

At the time of vomiting, the patient’s laboratory data demonstrated high levels of blood urea nitrogen (BUN) and creatinine (Cre). Uremic toxins may lead to the secretion of proinflammatory cytokines into the circulation of CKD patients. We therefore regularly measured serum proinflammatory cytokine and chemokine levels in the present patient.

## Case report

A 39-year-old woman had a twin pregnancy. At 34 weeks of gestation, she underwent a cesarean section, and dichorionic diamniotic male twins were born. One twin’s (the patient’s) birth weight was 1400 g. He required a high-flow nasal cannula for 8 days and had delayed circulatory failure for 9 days. At birth, abdominal ultrasonography displayed left MCDK and a normoechogenic right kidney of normal size. At 64 days, his weight was 2,230 g and he was discharged without oxygen. At the time of discharge, abdominal ultrasonography displayed an atrophic left kidney with few cysts and a slightly hyperechogenic right kidney. General laboratory examinations did not demonstrate any clinical abnormalities. The other twin’s birth weight was 1500 g. He required invasive ventilation for one day, and he was weaned off all respirqatory support at 7 days. He was discharged at 47 days with a weight of 2958 g.

**Fig. 1:**
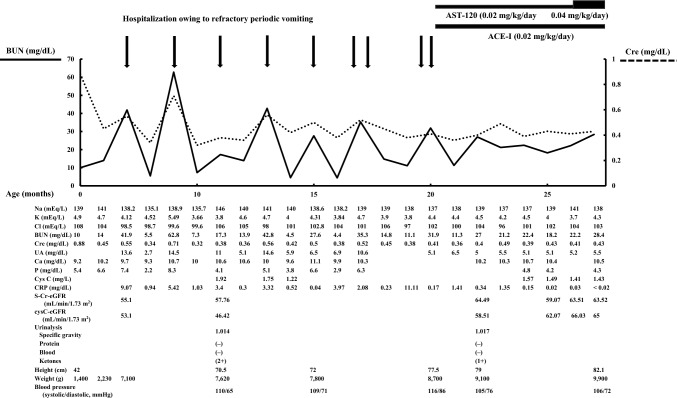
Clinical course of the patient. After combination therapy, the patient’s frequent hospitalizations owing to refractory periodic vomiting ceased, and his blood pressure decreased

After discharge, from 6 months after birth, the patient was frequently hospitalized owing to refractory periodic vomiting (Fig. 1). The refractory periodic vomiting started suddenly without any warning signs and he needed an intravenous drip every time. At the time of vomiting, his laboratory data demonstrated high levels of BUN (41.9–62.8 mg/dL) and Cre (0.55–0.71 mg/dL). At 9 months old, he was referred to our hospital. Abdominal ultrasonography displayed only traces of previous cysts in the left kidney and a hyperechogenic right kidney of appropriate size for his revised age. The patient was diagnosed with stage 3a CKD in accordance with the Evidence-Based Clinical Practice Guidelines for CKD 2023 [6].

At 14 months old, renal DTPA scintigraphy with Tc-99 m dimercaptosuccinic acid displayed differential uptake rates of 0% and 23.4% for the left and right kidney, respectively, suggesting that the left kidney was nonfunctional.

We suspected that the patient had some underlying diseases or conditions, such as gastric volvulus and gastroesophageal reflux disease, so we performed upper gastrointestinal X-ray using the contrast agent Gastrografin, and upper gastrointestinal endoscopy. The results were normal, and gastric volvulus and gastroesophageal reflux were not detected. Although we had suspected congenital inborn errors of immunity or metabolism, the findings were normal. His electroencephalogram was age appropriate, without any signs of neurological disease. Therefore, the cause of his refractory periodic vomiting remains unknown.

At 20 months old, the patient presented with high blood pressure (116/86 mmHg). Abdominal MRI displayed no signs of adrenal enlargement, and brain MRI displayed no abnormal signs or microvascular lesions. These results suggested that the patient had renal parenchymal hypertension owing to CKD. His hypertension was diagnosed as stage 2, in accordance with the 2017 American Academy of Pediatrics guidelines [7].

The patient began treatment with ACE-I and AST-120 at 20 months old. Subsequently, frequent hospitalization owing to refractory periodic vomiting ceased, and his blood pressure decreased from stage 2 to elevated blood pressure (105/76 mmHg). The patient’s S-Cr-eGFR and cys C-eGFR levels had not worsened since the start of treatment (Fig. 1).

We regularly measured many serum cytokines/chemokines (IL-1β, 2, 4, 6, 8, 10, 12, and 17, G-CSF, GM-CSF, IFN-γ, TNF-α, CXCL1, 2, 5, 6, 8, 9, 11, 12, 13, and 16, CCL1, 2, 3, 7, 8, 11, 13, 15, 17, 19, 20, 21, 22, 23, 24, 25, 26, and 27, and CX3CL1) using the 40- and 27-Plex Panel (Bio-Rad Laboratories, Tokyo, Japan).

Although the serum levels of proinflammatory cytokines (IFN-γ, IL-1β, IL-6, and TNF-α) remained low (Table 1), and many chemokines remained unchanged (Table 2), the levels of the chemokines CX3CL-1 and CCL20 were found to decrease with age (Table 1).

**Table 1:** Age-dependent changes in levels of proinflammatory cytokines (IFN-γ, IL-1β, IL-6, and TNF-α) and chemokines (CX3CL1 and CCL20) of the patient

Age (months)	IFN-γ	IL-1β	IL-6	TNF-α	CX3CL1	CCL20
11	86.49	0.47	2.52	104.48	2148	115.68
19	58.14	0.47	5.33	116.28	545	80.61
21	60.07	0.71	2.59	104.03	0	55.72
23	65.81	0.69	5.53	102.49	0	0

Values are shown as pg/mL

**Table 2:** Age-dependent changes in the levels of various other cytokines and chemokines of the patient

Age (months)	IL-2	IL-4	IL-8	IL-10	IL-12	IL-17	G-CSF	GM-CSF
11	1.62	2.41	19.63	4.38	8.72	6.22	118.55	0.29
19	0.32	2.16	26.66	8.38	5.11	5.61	138.17	0.11
21	1.69	2.07	19.99	1.45	0	3.78	185.82	0
23	1.62	1.72	12.18	0	0	4.13	158.11	0

Age (months)	CXCL1	CXCL2	CXCL5	CXCL6	CXCL8	CXCL9	CXCL11	CXCL12	CXCL13	CXCL16
11	5718	12,559	10,850	1129	96.43	1064	1754	91,791	2958	5719
19	6256	19,329	9439	1497	131.37	1337	2622	64,572	3637	4747
21	5951	15,329	7976	1411	112.66	1268	1972	52,277	2851	3219
23	4874	20,537	6263	1023	92.2	2735	1744	50,375	1947	3205

Age (months)	CCL1	CCL2	CCL3	CCL7	CCL8	CCL11	CCL13	CCL15	CCL17	CCL19
11	3265	591.13	40.32	1827	1175	3109	1509	34,547	3911	3262
19	3965	598.2	40.85	4655	825	3828	1839	19,762	4860	4194
21	3680	497.21	35.98	4012	632	3706	1603	16,494	6009	5191
23	2783	466.03	28.54	1827	1159	3041	1155	14,860	3744	3457

Age (months)	CCL21	CCL22	CCL23	CCL24	CCL25	CCL26	CCL27
11	10,91,248	28,157	2705	6447	34,760	7333	10,365
19	27,13,382	20,358	2410	6072	27,012	8483	19,276
21	29,29,514	20,939	2392	6333	22,980	8126	18,629
23	27,48,697	19,997	1909	5631	16,871	5460	10,398

Values are shown as pg/mL

At 9 months old, the patient had not achieved sitting without help. At 18 months old, he was able to walk without support and learn more words. His height, weight, and mental development are also catching up smoothly. On abdominal ultrasonography, his hyperechogenic right kidney had become normoechogenic, with an appropriate size for his revised age. At 28 months old, the patient was asymptomatic, although his laboratory data demonstrated high levels of BUN (28.4 mg/dL). His AST-120 dose was increased from 0.02 mg/kg/day to 0.04 mg/kg/day.

## Discussion

The cause of the patient’s refractory periodic vomiting remains unclear. At first, we suspected that the refractory periodic vomiting was owing to uremia; however, refractory periodic vomiting is unlikely to occur repeatedly as a symptom of uremia in CKD stage 3a patients. We hence investigated the possibility that the patient had some underlying diseases or conditions, such as digestive, immune, metabolic, or neurological diseases. From his clinical symptoms, cyclic vomiting syndrome was mainly suspected. However, this typically occurs in preschool or early school-age children, and it is difficult to diagnose cyclic vomiting syndrome in infants. Our present patient had CAKUT owing to left MCDK and a hyperechogenic right kidney. There is hence a possibility that he had impaired kidney function owing to CAKUT, which led to refractory periodic vomiting and dehydration.

In Japan, AST-120 and ACE-I are not strongly recommended for children with CKD, as an association between ACE-I treatment and acute kidney injury in CKD patients has been reported [5]. To prevent acute kidney injury, the patient was prohibited from taking oral ACE-I when he was not well, such as if he had a fever, cold, other infectious diseases, etc. In his clinical course, the combination therapy of AST-120 and ACE-I was effective for his refractory periodic vomiting and hypertension associated with CKD (Fig. 1).

We regularly measured the serum levels of various cytokines and chemokines in this patient. When his levels of BUN and Cre were high and he had refractory periodic vomiting, the levels of proinflammatory cytokines (IFN-γ, IL-1β, IL-6, and TNF-α) were suspected to be high. However, the serum proinflammatory cytokine levels of this patient were constantly low and unchanged. This suggested that proinflammatory cytokines might not be associated with the pathophysiology of refractory periodic vomiting.

Chemokines have traditionally received less attention than proinflammatory and anti-inflammatory cytokines; however, they have increasingly been reported to be involved in various diseases as diagnostic and/or prognostic biomarkers [8, 9]. Chemokines are small cytokine proteins that are involved in various physiological and immunological functions, and act as potent attractants that recruit immune cells to sites of inflammation. They are structurally divided into four classes based on the arrangement of their cysteine residues in the N-terminal region, namely, the CCL, CXCL, CX3CL, and XCL chemokines.

The function of CCL chemokines is to attract mononuclear cells to sites of chronic inflammation. The function of CXCL chemokines is to attract polymorphonuclear leukocytes to sites of acute inflammation. CX3CL chemokines are expressed on activated endothelial cells that are responsible for leukocyte adhesion and migration. The function of XCL chemokines is to attract specific subsets of T cells and natural killer cells [9].

We focused on the chemokines CX3CL1 and CCL20, as their levels were decreased with age in our patient, although the normal range of many chemokines in a 1-year-old infant is unclear. When the patient was 19 months old, which was before starting treatment (at 20 months old), the levels of these two chemokines were decreasing and improvement in his renal function was recognized at the same time. The improvement in his refractory periodic vomiting was suspected to be the result of an age-dependent phenomenon rather than owing to the treatment.

The CX3CL1 chemokine is also referred to as fractalkine. Gong Q et al. demonstrated that fractalkine deficiency suppresses macrophage activation and reduces lipopolysaccharide-induced acute kidney injury in mice, although it was unable to compare human acute kidney injury [10]. Recently, targeting CX3CL1 and its receptor CX3CR1 has recently been considered as a potential therapy for CKD [11].

CCL20 is expressed in tubular, endothelial, and interstitial cells, and has been reported to be upregulated in human kidneys with acute kidney injury. Urinary CCL20 was also increased in patients with acute kidney injury and was associated with renal severity [12].

The normal range of CXC3CL1 and CCL2 levels in a 1-year-old infant remains unclear; however, their levels appear to increase with the deterioration of renal function. Therefore, the decrease in CXC3CL1 and CCL2 levels observed in this patient was considered to be important. The association between AST-120, ACE-I, and chemokines remains unclear, and further investigation is needed in the future.

There are several limitations to this study. It is necessary to increase the number of cases and to measure cytokine/chemokine levels, not only in serum but also in urine samples, to confirm our present results. Furthermore, it is difficult to infer the association between chemokine levels and renal function from our present results. However, some specific chemokines might be diagnostic and/or prognostic biomarkers for CKD and should be investigated in future studies.
